# A phase 2 randomized controlled dose-ranging trial of recombinant pertussis booster vaccines containing genetically inactivated pertussis toxin in pregnant women

**DOI:** 10.1016/j.vaccine.2023.06.001

**Published:** 2023-07-12

**Authors:** Thanyawee Puthanakit, Kulkanya Chokephaibulkit, Surasith Chaithongwongwatthana, Niranjan Bhat, Yuxiao Tang, Suvaporn Anugulruengkitt, Chenchit Chayachinda, Sanitra Anuwutnavin, Keswadee Lapphra, Supattra Rungmaitree, Monta Tawan, Indah Andi-Lolo, Renee Holt, Librada Fortuna, Chawanee Kerdsomboon, Vilasinee Yuwaree, Souad Mansouri, Pham Hong Thai, Bruce L. Innis

**Affiliations:** a Department of Pediatrics, Faculty of Medicine and Center of Excellence in Pediatric Infectious Diseases and Vaccines, Chulalongkorn University, Rama IV Road, Bangkok 10330, Thailand; b Department of Obstetrics and Gynecology, Faculty of Medicine, Chulalongkorn University, Rama IV Road, Bangkok 10330, Thailand; c Siriraj Institute of Clinical Research (SICRES) Faculty of Medicine Siriraj Hospital, Mahidol University, 2 Wanglang Road, Bangkok 10700, Thailand; d Department of Pediatrics, Faculty of Medicine Siriraj Hospital, Mahidol University, 2 Wanglang Road, Bangkok 10700, Thailand; e Department of Obstetrics & Gynaecology, Faculty of Medicine Siriraj Hospital, Mahidol University, 2 Wanglang Road, Bangkok 10700, Thailand; f PATH, 2201 Westlake Avenue, Suite 200, Seattle, WA 98121, USA; g BioNet-Asia Co., Ltd., 19 Soi Udomsuk 37, Sukhumvit 103 Road, Bangjak, Prakanong, Bangkok 10260, Thailand

**Keywords:** Pertussis, Genetically inactivated, Maternal immunization, Recombinant pertussis vaccine, Safety, Immunogenicity

## Abstract

**Introduction:**

Despite a decrease in infections caused by *Bordetella pertussis* due to COVID-19 pandemic, booster vaccination of pregnant women is still recommended to protect newborns. Highly immunogenic vaccines containing genetically inactivated pertussis toxin (PT_gen_) and filamentous hemagglutinin (FHA) may generate comparable anti-PT antibody concentrations, even at lower doses, to chemically inactivated acellular pertussis vaccines (Tdap_chem_) shown effective for maternal immunization.

**Methods:**

This phase 2 randomized, observer-blind, active-controlled non-inferiority trial was conducted in healthy Thai pregnant women randomly assigned to receive one dose of low-dose recombinant pertussis-only vaccine containing 1 µg PT_gen_ and 1 µg FHA (ap1_gen_), or tetanus, reduced-dose diphtheria combined with ap1_gen_ (Tdap1_gen_), or combined with 2 µg PT_gen_ and 5 µg FHA (Tdap2_gen_), or with 5 µg PT_gen_ and 5 µg FHA (TdaP5_gen_, Boostagen®) or comparator containing 8 µg of chemically inactivated pertussis toxoid, 8 µg FHA, and 2.5 µg pertactin (Boostrix™, Tdap8_chem_). Blood was collected at Day 0 and Day 28 post-vaccination. The non-inferiority of the study vaccines was assessed based on anti-PT IgG antibody levels on Day 28 pooled with results from a similarly structured previous trial in non-pregnant women.

**Results:**

400 healthy pregnant women received one dose of vaccine. Combined with data from 250 non-pregnant women, all study vaccines containing PT_gen_ were non-inferior to comparator vaccine (Tdap8_chem_). Both ap1_gen_ and TdaP5_gen_ vaccines could be considered to have superior immunogenicity to Tdap8_chem_. Local and systemic solicited reactions were similar among all vaccine groups.

**Conclusions:**

Vaccine formulations containing PT_gen_ were safe and immunogenic in pregnant women. The ap1_gen_ vaccine, with the lowest cost and reactogenicity, may be suitable for use in pregnant women when diphtheria and tetanus toxoids are not needed.

This study is registered in the Thai Clinical Trial Registry (www.clinicaltrials.in.th), number TCTR20180725004.

## Highlights

• Recombinant acellular pertussis vaccine is safe and immunogenic in pregnant women.
• Vaccine formulations containing PT_gen_ were non-inferior to comparator vaccine.
• Low-dose ap1_gen_ is good alternative for pregnant women when DT and TT are not needed.

## 1 Introduction

Pertussis, a respiratory disease caused by the bacterium *Bordetella pertussis*, continues to be a significant worldwide health problem [1]. Although pertussis infection can cause disease at any age, the disease is most severe in infants and newborns [2], [3] with the highest case fatality rates between birth and 6 to 8 weeks of age [4]. Maternal immunization boosts the concentration of maternal antibodies that can be transferred across the placenta to directly protect infants too young to be immunized against tetanus, diphtheria and pertussis [5].

Several studies had already demonstrated the immunogenicity of the “pertussis vaccination during pregnancy” vaccination strategy besides the safety and effectiveness of vaccinating pregnant women with tetanus, diphtheria, acellular pertussis (Tdap) vaccine [6], [7], [8], [9]. In addition, Moro and colleagues (2016) had observed no new or unexpected vaccine-related adverse events (AE) in pregnant women who received Tdap vaccines following routine recommendations for maternal Tdap vaccination [10].

Vaccination of pregnant women with Tdap vaccine in the second or third trimester of pregnancy up to 15 days before delivery has been recommended by the World Health Organization [2].

A vaccine containing recombinant genetically inactivated pertussis PT (PT_gen_) and filamentous hemagglutinin (FHA) alone (aP5_gen_) or combined with tetanus and reduced-dose diphtheria toxoids (TdaP5_gen_) was developed and licensed for immunization of individuals aged 11 years and older in Thailand and in Singapore. Considering the high immunogenicity of PT_gen_, we postulated that lower-dose and therefore cost-saving formulations of the licensed recombinant pertussis vaccines may be suitable candidates for maternal pertussis immunization in low-to-middle income countries if shown to induce sufficiently high PT antibody levels that may be assumed to protect newborns against severe pertussis in the first months of life. We obtained initial evidence of acceptable safety and immunogenicity in women of childbearing age in a randomized controlled phase 2 trial of lower dose investigational formulations of these new generation recombinant pertussis vaccines [11], and through a post-licensure safety study of aP5_gen_ and TdaP5_gen_ vaccination in 1,778 pregnant women [12]. In the present phase 2 trial, we extended the investigation of these novel PT_gen_ formulations by comparing serological responses and safety in pregnant women following vaccination across several low-dose formulations versus the comparator vaccine.

## 2 Methods

### 2.1 Study design and participants

**Fig. 1:**
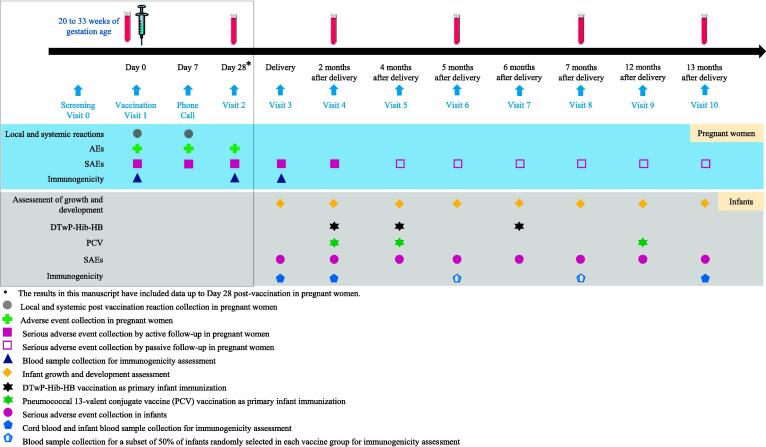
Study design. Pregnant women were given one dose of study vaccine between ≥20 weeks and <33 weeks gestational age. Local and systemic reactions were collected within 7 days after vaccination. The safety assessment was performed by active follow-up until Visit 4 (2 months after delivery) and by passive follow-up until Visit 10 (13 months after delivery). The blood sample collection for immunogenicity assessment was performed at Visit 1 (before vaccination), Visit 2 (28 days after vaccination) and Visit 3 (delivery). The growth and development assessment and safety assessment for infants were performed at Visit 3 (delivery) until Visit 10 (13 months of infant age). Infants received DTwP-Hib-HB vaccine at 2, 4 and 6 months of age and Pneumococcal 13-valent conjugate vaccine (PCV) at 2, 4 and 12 months of age as primary infant immunization. The cord blood sample at delivery and infant blood sample at Visit 4 (2 months of age), Visit 6 (5 months of age), Visit 8 (7 months of age) and Visit 10 (13 months of age) were collected for immunogenicity assessment.

The design of this phase 2, dual-site trial was based on a previous randomized observer-blind trial involving 250 women of childbearing age (50 participants per vaccine group) [11] in which the same investigational and comparator vaccines, as described below, were evaluated. The study vaccines were given as single dose to pregnant women (400 participants: 80 per vaccine group) during the second or third trimester of pregnancy at 20–33 weeks of gestation. The vaccination, frequency of study visits, and the safety and immunogenicity assessments are shown in Fig. 1.

Healthy pregnant women, 18 to 40 years old with no underlying chronic medical conditions, and an uncomplicated singleton pregnancy at ≥20 weeks and <33 weeks gestational age as confirmed by ultrasound were invited to participate. Women who had previously received diphtheria or tetanus or pertussis-containing vaccine within 1 year prior to enrolment were excluded (complete inclusion/exclusion criteria in Appendix Table 1).

The trial was conducted according to the guidelines of the International Council for Harmonisation of Technical Requirements for Pharmaceuticals for Human Use (ICH) and Good Clinical Practice (GCP), the Declaration of Helsinki, and local ethical guidelines. Ethical approval was obtained from the Institutional Review Boards of the Faculty of Medicine Siriraj Hospital at Mahidol University, Faculty of Medicine at Chulalongkorn University, Bangkok, Thailand and Western Institutional Review Board (now known as WIRB-Copernicus Group), Washington, USA. Written informed consent was obtained from all participants before recruitment into the study.

### 2.2 Randomization and masking

Participants were block-randomized in a 1:1:1:1:1 ratio per study site (80 participants per vaccine group; 40 per study site) to receive one dose (0.5 mL intramuscular injection) of either ap1_gen_, Tdap1_gen_, Tdap2_gen_, TdaP5_gen_ (Boostagen®), or a comparator, Tdap8_chem_ (Boostrix™) according to a computer-generated (PROC PLAN, Statistical Analysis System (SAS®) version 9.4) randomization scheme. A randomization list in a sealed envelope was provided to non-blinded clinical research staff who were responsible for participant randomization for study vaccine assignment, vaccine preparation, administration, and accountability. All other research staff, study participants, and all laboratory personnel performing immunologic testing were blinded to the vaccine administered.

### 2.3 Study vaccines

The composition of the study vaccines is shown in Appendix Table 2. The recombinant acellular pertussis vaccines contain two pertussis components (PT_gen_ and filamentous hemagglutinin, FHA), whereas the chemically inactivated acellular pertussis comparator is a three-component vaccine containing PT_chem_, FHA, and pertactin (PRN). The study evaluated the stand-alone recombinant acellular pertussis vaccine (ap_gen_, pertussis-only) and trivalent (combination) vaccines containing tetanus, diphtheria, in addition to recombinant acellular pertussis vaccines (Tdap or TdaP). Uppercase letters ‘T’ and ‘P’ in these abbreviations mean the vaccine has full-strength doses of tetanus and pertussis. The lowercase letters ‘d’ and ‘p’ refer to reduced-dose diphtheria and pertussis.

The recombinant acellular pertussis vaccines were developed and manufactured at BioNet-Asia, Ayutthaya, Thailand, namely: low-dose recombinant acellular pertussis-only vaccine, ap1_gen_ containing PT_gen_ (1 µg) and FHA (1 µg); Tdap1_gen_ containing tetanus toxoid (TT) (7.5 Lf), reduced-dose diphtheria toxoid (DT) (2.0 Lf), PT_gen_ (1 µg) and FHA (1 µg); Tdap2_gen_ containing TT (7.5 Lf), DT (2.0 Lf), PT_gen_ (2 µg) and FHA (5 µg); TdaP5_gen_ (Boostagen®) containing TT (7.5 Lf), DT (2.0 Lf), PT_gen_ (5 µg) and FHA (5 µg). The comparator vaccine, Boostrix™ (Tdap8_chem_) was manufactured by GlaxoSmithKline (GSK), Belgium, and contains TT (5 Lf), DT (2.5 Lf), chemically inactivated pertussis toxin (PT_chem_) (8 μg), FHA (8 µg), and PRN (2.5 µg).

#### 2.3.1 Immunogenicity assessments

Venous blood samples (5 mL) were obtained before vaccine administration (Day 0; baseline), and 28 days after vaccination (Day 28). It was then processed for serum separation on the same day. The blood sample was centrifuged at 1300g for 15 minutes at room temperature. The serum sample was collected and stored at −20 ⁰C and below prior to analysis. Concentrations of serum IgG antibody against PT_gen_ and FHA were measured using an in-house validated indirect enzyme-linked immunosorbent assay (ELISA), and concentrations were expressed in IU/mL, calibrated to the WHO International Standard Pertussis Antiserum (Human) 06/140. The WHO Reference Reagent Pertussis Antiserum (Human) 06/142 (NIBSC, UK) was used as the positive control [13]. Serum TT- and DT-specific IgG concentrations were measured using validated commercially available ELISA kits (Serion ELISA classic Diphtheria IgG kit, ESR130G; and Serion ELISA Classic Tetanus IgG kit, ESR108G, Virion/Serion, Germany), and concentrations were expressed in IU/mL. Functional anti-PT antibody titers were assessed in a randomly selected 30% subset of study participants using a validated Chinese hamster ovary (CHO) cell pertussis toxin neutralization assay and JNIH-5 (NIBSC) as the source of pertussis toxin, as described previously [14], [15], with titer reported as IU/mL on the basis of the relative activity of the WHO International Standard Pertussis Antiserum (Human) 06/140 [13]. The lower limits of quantification (LLOQ) (assay cut-off) were 5 IU/mL for PT-IgG, 1 IU/mL for FHA-IgG and 0.05 IU/mL for DT-IgG and TT-IgG. Antibody concentrations below the assay cut-offs (or LLOQ) were given arbitrary values of half the assay cut-offs.

#### 2.3.2 Safety assessment

The schedule of safety and immunogenicity assessments is shown in Fig. 1. Participants were monitored for 30 minutes post-immunization for any immediate reactions. Diary cards were distributed on the day of vaccination to record solicited adverse events (AEs) at the injection site (pain, redness, swelling, induration), or systemic AEs (fever, headache, fatigue, malaise, arthralgia, chills, myalgia, nausea, vomiting) for 7 days after vaccination. Unsolicited AEs, medically attended adverse events (MAAEs) and serious adverse events (SAEs) were collected until study end. However, in this paper, MAAEs and SAEs were presented up to 28 days after vaccination. AEs were assessed by the investigator for severity using the Division of AIDS (DAIDS) Table for grading the severity of adult and pediatric adverse events of the US National Institute of Health, 2017 [16], and causality by the study vaccine according to ICH guidelines [17] and protocol-specified safety considerations.

### 2.4 Outcomes

The primary outcome was the assessment of non-inferior immunogenicity of each study group (ap1_gen_, Tdap1_gen_, Tdap2_gen_, or TdaP5_gen_) to the comparator group (Tdap8_chem_) based on the geometric mean of anti-PT antibody concentration (GMC) on Day 28 after vaccination in the pooled per-protocol population of pregnant women and women of childbearing age. The secondary outcomes for immunogenicity included the following comparisons of each study group (ap1_gen_, Tdap1_gen_, Tdap2_gen_, or TdaP5_gen_) to the comparator group, Tdap8_chem_ in pregnant women only: (i) comparison of GMC of anti-PT and anti-FHA antibodies and geometric mean titer (GMT) of PT neutralizing antibody; (ii) comparison of seroconversion rate of anti-PT, anti-FHA antibodies and PT neutralizing antibody; (iii) comparison of seroprotection rate and GMC of anti-DT and anti-TT antibody. For anti-PT, anti-FHA and PT neutralizing antibodies, seroconversion was defined as an antibody concentration/titer increase of four-fold or more from baseline to Day 28. Seroprotection for anti-DT and anti-TT antibody was defined as an antibody concentration ≥ 0.1 IU/mL at any time point. The secondary outcomes for safety were (i) percentage of participants with solicited local and systemic AEs reported within seven days after vaccination (ii) percentage of participants with unsolicited AEs, MAAEs, and SAEs reported through 28 days following vaccination.

### 2.5 Statistical analysis

The sample size was calculated based on non-inferiority test with a significance level of 0.00625 for the primary objective after a Bonferroni adjustment. In order to have sufficient sample size to conduct the primary analysis, we pooled both pregnant women (80 enrolled per group; 400 in total) recruited in the current study and non-pregnant women of childbearing age from the previous study (50 enrolled per group; 250 in total) [11] for a total of 130 women per group in the pooled population.

The power to show comparability of immune response in terms of anti-PT GMCs 28 days after a single dose with study vaccine versus comparator was calculated using a one-sided two-sample *t*-test with a significance level of 0.00625. Assuming the standard deviation of log_10_ anti-PT ELISA antibody concentration was 0.6 in both study vaccine and comparator groups and the true anti-PT GMC ratio between the two groups was 1.0, with 117 evaluable participants per group in the pooled population (assuming an attrition rate of 10%), and a non-inferiority margin of 0.5, the study had 91% power to detect non-inferiority of ap1_gen_/Tdap1_gen_/Tdap2_gen_/TdaP5_gen_ versus the Tdap8_chem_ group.

With 80 pregnant women vaccinated per group, the present study had a 95% probability to detect at least one SAE if the true incidence is 3.7%. Conversely, if no SAEs were observed in 80 vaccine recipients, the study would be able to rule out SAEs occurring at a rate of approximately 4.6% or above, based on the upper bounds of the 95% CI.

To assess the primary outcome, GMC of anti-PT antibody at 28 days following immunization in pregnant women and women of childbearing age [11] were computed for each vaccine group along with its two-sided 95% CI, by exponentiating the corresponding log-transformed mean and its 95% CI limit. The ratio of the GMC in each of the ap1_gen_/Tdap1_gen_/Tdap2_gen_/TdaP5_gen_ groups to that in the Tdap8_chem_ group and a two-sided 98.75% CI for the ratio were provided. The log_10_-transformed concentrations were used to construct a mean difference between the two vaccine groups and its two-sided 98.75% CI using analysis of covariance (ANCOVA). The log_10_-transformed baseline concentrations and an indicator of study populations were included as covariates. Other variables such as age, study site, and gestational age were evaluated for inclusion in the ANCOVA model as covariates. Interaction terms among selected covariates were evaluated for inclusion as well. The mean difference and corresponding 98.75% CI limits were exponentiated to obtain the GMC ratio and the corresponding 98.75% CI. Non-inferiority was demonstrated if the lower confidence limit of the 98.75% CI of the GMC ratio (GMC_S_/GMC_C_) was larger than 0.5 (non-inferiority margin) (where s refers to each study vaccine group and c refers to the comparator group).

The secondary endpoints, GMC of anti-PT, anti-FHA, anti-tetanus and anti-diphtheria, and GMT of PT neutralizing antibody at baseline and 28 days following immunization in pregnant women, were calculated for each vaccine group along with two-sided 95% CI. The ratio of the GMC or GMT in each of the ap1_gen_/Tdap1_gen_/Tdap2_gen_/TdaP5_gen_ groups to that in the Tdap8_chem_ group and a two-sided 95% CI for the ratio were obtained using methods similar to those outlined above. Seroconversion rates for anti-PT and anti-FHA antibody and PT neutralizing antibody, and seroprotection rates for anti-DT and anti-TT antibody were computed along with the corresponding exact two-sided 95% CI based on the Clopper-Pearson method for each vaccine group. The differences in the seroconversion rates between each of ap1_gen_/Tdap1_gen_/Tdap2_gen_/TdaP5_gen_ groups and the Tdap8_chem_ group were calculated along with the two-sided 95% CI obtained by the Miettinen and Nurminen method. All immunogenicity analyses were done in both full analysis (FA) and per protocol (PP) populations with the PP population as the primary analysis population.

For safety analysis, the proportion of participants with safety endpoints at different study time points along with an exact two-sided 95% CI were calculated using the Clopper-Pearson method. A pair-wise comparison in the percentage of participants with safety endpoints between each of the ap1_gen_/Tdap1_gen_/Tdap2_gen_/TdaP5_gen_ groups and the Tdap8_chem_ group was performed using Fisher’s exact test. All safety analyses were done in the safety population.

All statistical analyses were performed using SAS® software version 9.4.

## 3 Results

This paper reports the findings from the initial phase of the study up to Day 28. The immunogenicity results from the PP population are presented. The disposition of the pregnant women meeting the protocol requirements is shown in Fig. 2a. Between January 4, 2019, and October 10, 2019, a total of 412 pregnant women were screened, of whom 400 (200 from each study site) were enrolled and randomized into five vaccine groups (80 pregnant women per vaccine group). All enrolled pregnant women were included in the safety analysis (Safety population, N=400). The PP population for immunogenicity analysis included 394 (six pregnant women were excluded as one subject did not have blood sample collection and five had at least one major protocol deviation that might affect the immunogenicity results).

**Fig. 2a:**
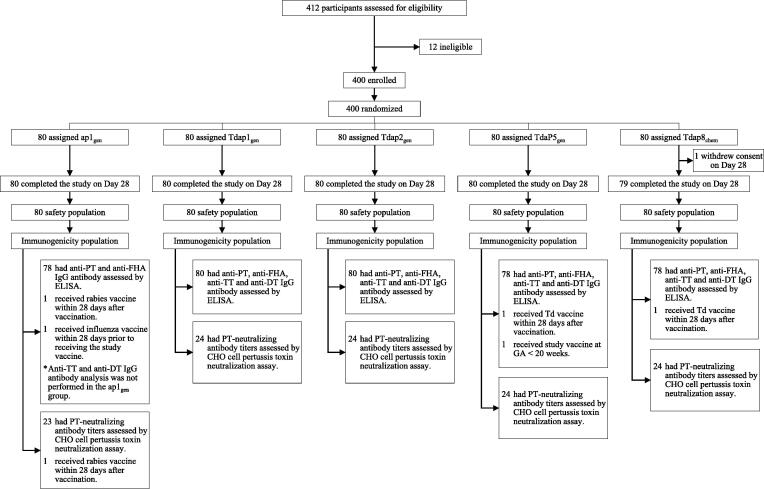
Trial profile (pregnant women).

To assess the primary objective of the present study, we added the PP population from women of childbearing age (250 women consisting of 50 in each vaccine group) to the PP population of pregnant women (394 pregnant women). Hence, the pooled population included 644 participants in total (Fig. 2b). For other analyses outside the primary analysis (non-inferiority test), only data from pregnant women were used.

**Fig. 2b:**
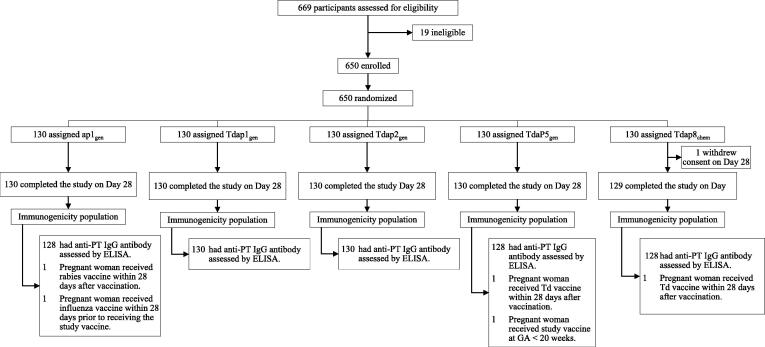
Pooled population of pregnant women and non-pregnant women of childbearing age.

Demographic characteristics (age, ethnicity, height, and body weight) at baseline were similar for participants in all vaccine groups (Table 2).

**Table 1:** Baseline demographic characteristics.

	**Pooled population of pregnant women and** **non-pregnant women of childbearing age**						**Pregnant women**					
	**ap1_gen_** **(n = 130)**	**Tdap1_gen_** **(n = 130)**	**Tdap2_gen_** **(n = 130)**	**TdaP5_gen_** **(n = 130)**	**Tdap8_chem_** **(n = 130)**	**Total** **(n = 650)**	**ap1_gen_** **(n = 80)**	**Tdap1_gen_** **(n = 80)**	**Tdap2_gen_** **(n = 80)**	**TdaP5_gen_** **(n = 80)**	**Tdap8_chem_** **(n = 80)**	**Total** **(n = 400)**
**Age, years**												
Mean (SD)	30.2 (5.0)	29.6 (5.6)	30.1 (5.5)	29.6 (5.7)	30.6 (5.3)	30.0 (5.4)	30.6 (4.7)	29.7 (5.1)	29.6 (6.0)	29.1 (5.7)	29.8 (5.2)	29.8 (5.3)
Median	30.0	29.0	30.0	30.5	31.0	30.0	31.0	29.0	30.0	30.0	30.0	30.0
Min/Max	18–39	18–39	18–39	18–39	19–39	18–39	19–39	18–39	18–39	19–39	19–39	18–39
**Ethnicity**, n (%)												
Asian	130 (100.0)	130 (100.0)	130 (100.0)	130 (100.0)	130 (100.0)	650 (100.0)	80 (100.0)	80 (100.0)	80 (100.0)	80 (100.0)	80 (100.0)	400 (100.0)
**Height, cm**												
Mean (SD)	158.4 (5.6)	159.1 (5.2)	158.5 (5.5)	158.8 (5.5)	159.8 (5.8)	158.9 (5.5)	158.3 (5.6)	159.5 (5.6)	158.8 (5.3)	159.4 (5.7)	160.0 (6.0)	159.2 (5.6)
Median	158.0	159.5	158.0	159.0	160.0	159.0	158.0	160.0	158.0	159.5	160.0	159.5
Min/Max	145–173	142–173	144–175	145–172	145–175	142–175	145–170	142–173	150–175	147–172	145–173	142–175
**Weight, kg**												
Mean (SD)	60.4 (12.1)	63.1 (12.1)	62.0 (12.8)	64.0 (14.1)	65.6 (13.2)	63.0 (13.0)	62.1 (10.9)	65.0 (10.7)	64.6 (12.0)	65.1 (12.5)	66.7 (12.3)	64.7 (11.7)
Median	58.8	62.8	59.7	61.6	63.6	61.4	62.1	64.7	62.8	62.8	65.2	63.6
Min/Max	39.3–96.1	41.2–101.1	38.8–101.4	37.7–111.6	42.6–113.5	37.7–113.5	40.3–90.5	45.4–94.9	41.8–101.4	37.7–101.9	45.5–113.5	37.7–113.5
Data are presented as mean (SD), median, min/max, n (%).												

### 3.1 Primary endpoint analysis of anti-PT antibody at Day 28 post-vaccination in the pooled population (pregnant women and non-pregnant women of childbearing age)

Adjusted GMC ratios of the anti-PT antibody of each group with the comparator, Tdap8_chem_ at Day 28 (Table 2) are as follows: 1.7 (98.75% CI 1.3–2.3), 0.9 (98.75% CI 0.7–1.3), 1.2 (98.75% CI 0.9–1.5), and 2.6 (98.75% CI 2.0–3.5) for ap1_gen_, Tdap1_gen_, Tdap2_gen_ and TdaP5_gen_, respectively. For the analysis of pooled data, the adjusted GMC was obtained by ANOVA adjusted for concentrations at baseline, study, and interaction between vaccine group and study. In this analysis, which included pregnant women and women of childbearing age, the primary outcome of non-inferiority was met. Moreover, the lower bound of the 98.75% CI of the adjusted GMC ratio was greater than one for ap1_gen_ and TdaP5_gen_, indicating that both vaccines could be considered superior to Tdap8_chem_ in inducing anti-PT antibody response.

**Table 2:** Anti-PT IgG in the pooled population of pregnant women and women of childbearing age, and antibody concentration/titers (anti-PT IgG, anti-FHA IgG and PT neutralizing antibody) in pregnant women at 28 days after vaccination.

**2A: Anti-PT IgG antibody concentration in the pooled population of pregnant women and women of childbearing age**															
	**GMC (95**% **CI) IU/mL**					**Adjusted GMC (95**% **CI)^1^IU/mL**					**Adjusted GMC Ratio (98.75**% **CI)^2^**				
	**ap1_gen_**	**Tdap1_gen_**	**Tdap2_gen_**	**TdaP5_gen_**	**Tdap8_chem_**	**ap1_gen_**	**Tdap1_gen_**	**Tdap2_gen_**	**TdaP5_gen_**	**Tdap8_chem_**	**ap1_gen_**	**Tdap1_gen_**	**Tdap2_gen_**	**TdaP5_gen_**	**Tdap8_chem_**
**N**	128	130	130	128	128	128	130	130	128	128	128	130	130	128	128
**Baseline**	6.0	5.6	4.7	5.0	6.4	**–**	**–**	**–**	**–**	**–**	**–**	**–**	**–**	**–**	**–**
	(5.0–7.3)	(4.7–6.5)	(4.0–5.4)	(4.2–5.9)	(5.3–7.7)	**–**	**–**	**–**	**–**	**–**	**–**	**–**	**–**	**–**	**–**
**Day 28**	91.7	49.5	56.2	133.7	56.1	93.3	50.1	61.7	141.3	53.7	1.7	0.9	1.2	2.6	**–**
	(73.9–113.9)	(42.8–57.2)	(46.5–67.9)	(111.4–160.5)	(48.2–65.4)	(79.6–110.0)	(43.1–59.4)	(52.3–72.1)	(119.0–164.3)	(45.7–63.1)	(1.3–2.3)	(0.7–1.3)	(0.9–1.5)	(2.0–3.5)	**–**

**2B: Antibodyconcentration/titers inpregnantwomen**															
	**Anti-PT IgG**					**Anti-FHA IgG**					**PT Neutralizing Antibody**				
	**ap1_gen_**	**Tdap1_gen_**	**Tdap2_gen_**	**TdaP5_gen_**	**Tdap8_chem_**	**ap1_gen_**	**Tdap1_gen_**	**Tdap2_gen_**	**TdaP5_gen_**	**Tdap8_chem_**	**ap1_gen_**	**Tdap1_gen_**	**Tdap2_gen_**	**TdaP5_gen_**	**Tdap8_chem_**
**Baseline, N**	78	80	80	78	78	78	80	80	78	78	23	24	24	24	24
**GMC or GMT** (IU/mL)	4.6	5.41	4.7	4.5	6.3	7.1	9.4	7.8	6.7	9.7	3.7	5.9	5.7	5.1	8.2
(95% CI)	(3.7–5.8)	(4.4–6.6)	(3.8–5.8)	(3.7–5.4)	(5.0–8.0)	(5.5–9.0)	(7.3–12.2)	(5.9–10.4)	(5.2–8.7)	(7.3–12.9)	(3.2–4.3)	(4.2–8.3)	(3.8–8.6)	(4.0–6.6)	(5.3–12.7)
**Day 28, N**	78	80	80	78	78	78	80	80	78	78	23	24	24	24	24
**GMC or GMT** (IU/mL)	65.3	44.7	53.8	125.9	51.2	89.4	60.5	112.0	112.4	195.3	40.2	36.5	55.1	107.0	48.2
(95% CI)	(49.0–87.0)	(38.2–52.3)	(42.9–67.4)	(100.6–157.5)	(42.5–61.7)	(77.9–102.6)	(50.8–72.1)	(90.5–138.7)	(94.9–133.3)	(157.6–242.0)	(20.3–79.5)	(26.6–50.0)	(36.2–84.0)	(66.4–172.5)	(32.4–71.6)
**Day 28/Baseline, N**	78	80	80	78	78	78	80	80	78	78	23	24	24	24	24
**GMFR**	14.1	8.3	11.4	28.3	8.1	12.7	6.4	14.3	16.7	20.1	10.9	6.2	9.6	21.0	5.9
(95% CI)	(10.7–18.6)	(6.8–10.1)	(9.2–14.2)	(22.7–35.2)	(6.5–10.2)	(10.2–15.7)	(5.3–7.9)	(11.0–18.6)	(13.4–20.8)	(15.5–26.2)	(5.9–20.3)	(4.6–8.4)	(6.6–14.2)	(14.3–30.9)	(4.0–8.6)
**Adjusted GMC or GMT Ratio^3^**	1.5*	**0.9**	**1.2**	2.9*	**–**	0.5*	0.3*	0.6*	0.7*	**–**	**2.6**	**0.9**	**1.4**	**3.0**	**–**
(98.75% CI or 95% CI)	**(1.0**–**2.1)**	**(0.7**–**1.3)**	**(0.8**–**1.7)**	**(2.0**–**4.1)**	**–**	**(0.4**–**0.6)**	**(0.3**–**0.4)**	**(0.5**–**0.8)**	**(0.5**–**0.8)**	**–**	**(1.3**–**5.3)**	**(0.5**–**1.6)**	**(0.8**–**2.4)**	**(1.7**–**5.3)**	**–**

^1^Adjusted GMC was obtained by ANOVA adjusted for concentrations at baseline, study, and an interaction between vaccine group and study. ^2^The ratio of adjusted GMC between each study group (ap1_gen_/Tdap1_gen_/Tdap2_gen_/TdaP5_gen_) and comparator group (Tdap8_chem_) for non-inferiority test. The lower limit of the 98.75% CI of the adjusted GMC ratio was compared to the non-inferiority margin of 0.5. ^3^PT: The ratio of adjusted GMC between each study group and comparator group with 98.75% CI was calculated based on ANCOVA model, adjusted for concentrations at baseline. FHA: The ratio of adjusted GMC between each study group and comparator group with 95% CI was calculated based on ANCOVA model, adjusted for concentrations at baseline and study site. PT neutralizing: The ratio of adjusted GMT between each study group and comparator group with 95% CI was calculated based on ANCOVA model, adjusted for concentrations at baseline and interaction between baseline and vaccine group. *The adjusted GMC or adjusted GMT between study group and comparator group is significantly different. Abbreviations: GMC, geometric mean concentration for PT and FHA; GMT, geometric mean titer for PT neutralizing; GMFR, geometric mean fold rise from baseline; CI, confidence interval.															

### 3.2 Other endpoint analysis at Day 28 post-vaccination in pregnant women only

At baseline, the GMC of anti-PT antibody was low and similar in all maternal vaccine groups. On Day 28, the anti-PT antibody GMC change from baseline was highest for the TdaP5_gen_ group followed by the ap1_gen_ group, and the lowest for the Tdap8_chem_ group. Day 28 adjusted GMC ratios between the study vaccines and comparator (Tdap8_chem_) ranged from 0.9 (98.75% CI 0.7–1.3, Tdap1_gen_) to 2.9 (98.75% CI 2.0–4.1, TdaP5_gen_). These results indicated that all study vaccines (ap1_gen_, Tdap1_gen_, Tdap2_gen_, and TdaP5_gen_) were at least as effective as the comparator (Tdap8_chem_) in inducing anti-PT antibody responses in pregnant women (Table 2).

Similarly, at baseline, the GMT of PT neutralizing antibody was low and similar in all groups. On Day 28, the GMT change from baseline was highest for the TdaP5_gen_ group followed by the ap1_gen_ group, and the lowest for the Tdap8_chem_ group. The Day 28 adjusted GMT ratio between the study vaccines and comparator (Tdap8_chem_) ranged from 0.9 (95% CI 0.5–1.6, Tdap1_gen_) to 3.0 (95% CI 1.7–5.3, TdaP5_gen_). Thus, all study vaccines containing PT_gen_ were shown to be at least as effective as the comparator vaccine (Tdap8_chem_) in inducing functionally active PT-neutralizing antibody in pregnant women (Table 2).

The GMC of anti-FHA antibody at baseline was similar in all groups. On Day 28, the change in GMC from baseline was highest in the Tdap8_chem_ group, followed by TdaP5_gen_, and lowest in the Tdap1_gen_ group. The Day 28 adjusted GMC ratios between the study vaccine and the comparator (Tdap8_chem_) ranged from 0.3 (95% CI 0.3–0.4, Tdap1_gen_) to 0.7 (95% CI 0.5–0.8, TdaP5_gen_) (Table 2).

**Fig. 3:**
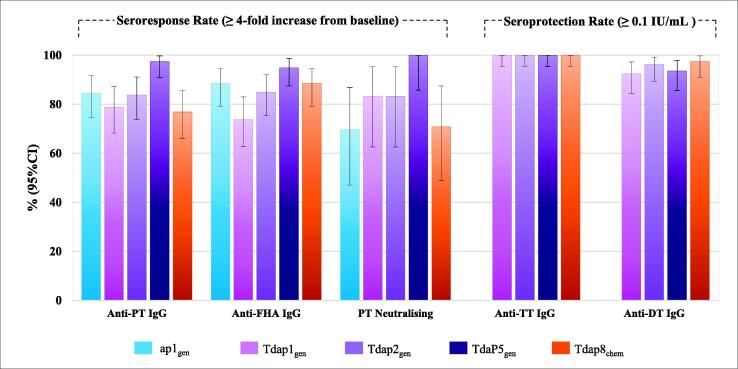
Anti-PT IgG, anti-FHA IgG, and PT-neutralizing antibody seroresponse rate and anti-TT IgG, anti-DT IgG seroprotection rate at 28 days after vaccination in pregnant women.

The proportion of pregnant women with ≥ 4-fold increase in antibody concentration to PT and FHA at Day 28 with respect to baseline (seroconversion rates) ranged from 76.9% to 97.4% for PT in Tdap8_chem_ and TdaP5_gen_ respectively, and from 73.8% to 94.9% for FHA in Tdap1_gen_ and TdaP5_gen_, respectively compared to 88.5% in Tdap8_chem_ (Fig. 3).

For PT neutralizing antibody, the seroconversion rate was 100% for TdaP5_gen_, and 69.6% for ap1_gen_, compared to 70.8% in the Tdap8_chem_ group (Fig. 3).

Reverse cumulative distribution curves (RCDCs) for anti-PT IgG, anti-FHA IgG, and PT neutralizing antibody at baseline and Day 28 after vaccination are shown in Appendix Fig. 1.

Participants vaccinated with the formulations containing tetanus (TT) and diphtheria (DT) toxoid antigens all showed satisfactory tetanus and diphtheria responses at Day 28, and the anti-TT and anti-DT antibody GMC changes from baseline were similar for all vaccines. The anti-TT and anti-DT responses elicited by the study vaccines were similar to those elicited by the comparator, Tdap8_chem_ except for the anti-TT antibody GMC in TdaP5_gen_ and the anti-DT antibody GMC in Tdap1_gen_ where the 95% CI of the adjusted GMC ratios did not include unity (Appendix Table 3).

The TT antibody concentration was ≥0.1 IU/mL (equated with seroprotection) in more than 96% of participants at baseline, rising to 100% by Day 28. For DT, the proportion of participants with antibody concentration ≥0.1 IU/mL (equated with seroprotection) was doubled from 46%, rising to 92% by Day 28 after vaccination (Fig. 3).

The solicited local and systemic reactions are presented in Appendix Table 4. Pain at the injection site was the most common solicited local reaction for all vaccine groups (72.5% for ap1_gen_, 80.0% for Tdap1_gen_ and Tdap2_gen_, 82.5% for TdaP5_gen_, and 88.8% for the comparator, Tdap8_chem_). Most of these cases were mild and lasted for a few days. Myalgia was the most commonly reported solicited systemic reaction in all vaccine groups. The proportion of pregnant women with myalgia was similar in Tdap2_gen_ and TdaP5_gen_ groups (36.3% and 35.0%, respectively) and Tdap8_chem_ (36.3%). However, fewer pregnant women reported myalgia in the ap1_gen_ (15.0%) and Tdap1_gen_ (25.0%) groups. Most systemic reactions were transient and mild or moderate in severity.

A total of 120 unsolicited AEs were reported by 20.3% of participants up to Day 28 following vaccination (Appendix Table 5). The incidence was similar among vaccine groups.

Overall, five SAEs (four cases of preterm labor and one case of vaginal bleeding) were reported up to Day 28 following vaccination. None of these SAEs were considered vaccine-related (Appendix Table 5).

## 4 Discussion

Our study focused on the induction by pertussis booster vaccines of anamnestic anti-PT response to obtain antibody levels sufficiently high to likely protect newborns against severe pertussis in the first months of life (data will be presented later in a separate publication). The antibody level of our study formulations has elicited similar or higher levels than the comparator in pregnant women at day 28 post-vaccination which was previously shown to protect infants in a study conducted by Amirthalingam et al (2014) [6].

Findings from this phase 2 trial indicate that recombinant genetically inactivated acellular pertussis vaccines may be suitable for use in immunizing pregnant women at a range of doses. Not only were they non-inferior in terms of immunogenicity to the chemically inactivated pertussis vaccine licensed for this use in the pooled population of pregnant women and women of childbearing age (Table 2), they also showed acceptable safety profiles (Table A4, Table A5). In addition, the ap1_gen_ and TdaP5_gen_ vaccines generated significantly higher antibody responses than the Tdap8_chem_ vaccine. The high immunogenicity of ap1_gen_, a low-dose stand-alone vaccine, is compatible with its use as a booster with an acceptable safety profile in pregnant women where the benefit of the additional tetanus and diphtheria toxoids is not required, analogous to the current use of aP5_gen_ (Pertagen®, a stand-alone recombinant acellular pertussis vaccine containing genetically inactivated PT and FHA).

In this study, we found that 57% of pregnant women were seronegative for PT-IgG antibody at baseline which is in line with another study conducted in Thai pregnant women [18]. The protection of newborn infants via the transfer of maternal antibodies through the placenta and breastmilk would be inadequate without maternal booster immunization [19], [20], [21]. The substantial enhancement of maternal antibodies by a single booster dose of any of the vaccines studied here was comparable to our previous findings with the same vaccines in women of childbearing age [11] and other recent studies of Tdap8_chem_ vaccination in pregnancy [22], [23], [24], [25]. Notably, we found no major difference in PT-IgG response and serum PT-neutralizing activity between vaccination during the second versus the third trimester of pregnancy when the responses were measured at 28 days after vaccination (Appendix Fig. 2).

For both seroresponse rates and GMC/GMT for PT antibody and PT neutralizing antibody, our study formulations have elicited similar or higher levels than the comparator. The higher levels were observed with Tdap2_gen_ and TdaP5_gen_ but more interestingly also with ap1_gen_, which contains only one microgram of PT_gen_.

For FHA antibody, ap1_gen_, Tdap2_gen_ and TdaP5_gen_ vaccines have induced similar or higher seroresponse rate than the comparator whereas it was found to be lower with Tdap1_gen_. Anti-FHA antibody GMCs are also lower in our study vaccines compared to Tdap8_chem_. However, the contribution of anti-FHA to protection against severe pertussis illness likely principally mediated by PT is expected to be limited [26]. This supports the fact that monocomponent vaccines containing only PT have been shown to be sufficient to protect infants from severe pertussis [27], [28]. In addition, an infant baboon model had shown that anti-PT alone was sufficient to prevent neonatal pertussis disease but not infection in animals intratracheally challenged with a large dose of pathogenic *Bordetella pertussis* [29]*.*

One limitation of this study in pregnant women is the small sample size. However, pooling of this study with the study in women of childbearing age provided adequate power for a non-inferiority comparison. The pooling was considered appropriate given the similar study methods, populations, immunogenicity testing in the same laboratory with the same validated assay, and adjustment of responses based on baseline titer, cohort, and treatment. Another limitation of this study is the fact that the immune system in pregnant women behave differently compared to non-pregnant women. By pooling the data, the effect of pregnancy on immune response will be decreased.

More information on pertussis immune response at birth and in infants will be needed before generalized conclusions ranking the responses to the different vaccine formulations can be drawn. Additional data from later stages/phases of the same study are forthcoming to assess the impact of the timing of maternal vaccination (second versus third trimester of pregnancy) on IgG placental transfer to the infant and to explore the impact of maternal antibodies on infant responses to primary immunizations, including to pneumococcal conjugate vaccine (PCV).

More extensive studies with the selected study vaccines should be then considered in pregnant women in other populations, in diverse countries, ethnicities, and social contexts.

## Funding

This work was funded by a grant from the Bill & Melinda Gates Foundation, Seattle, WA [grant number OPP1120084]. The findings and conclusions contained within are those of authors and do not reflect position or policies of the Bill & Melinda Gates Foundation.

## Author contribution

BLI, NB, SM, YT and PHT conceived and designed the study. KC, TP and SM implemented the study. TP and KC were the principal site investigators. SC, SAg, CC, SAw, KL, SR and MT conducted the study with support from LF, CK and VY. IAL and RH supported with project management of the funder. All authors contributed to data interpretation. All authors reviewed the drafts and approved the final version of the manuscript for submission.

## CRediT authorship contribution statement

**Thanyawee Puthanakit:** Supervision, Writing – review & editing. **Kulkanya Chokephaibulkit:** Supervision, Writing – review & editing. **Surasith Chaithongwongwatthana:** Investigation, Writing – review & editing. **Niranjan Bhat:** Conceptualization, Methodology, Writing – review & editing. **Yuxiao Tang:** Conceptualization, Methodology, Writing – review & editing. **Suvaporn Anugulruengkitt:** Investigation, Writing – review & editing. **Chenchit Chayachinda:** Investigation, Writing – review & editing. **Sanitra Anuwutnavin:** Investigation, Writing – review & editing. **Keswadee Lapphra:** Investigation, Writing – review & editing. **Supattra Rungmaitree:** Investigation. **Monta Tawan:** Investigation. **Indah Andi-Lolo:** Writing – review & editing. **Renee Holt:** Writing – review & editing. **Librada Fortuna:** Investigation, Validation, Writing – review & editing. **Chawanee Kerdsomboon:** Investigation, Validation, Writing – review & editing, Visualization. **Vilasinee Yuwaree:** Investigation, Writing – review & editing. **Souad Mansouri:** Conceptualization, Methodology, Supervision, Writing – review & editing. **Pham Hong Thai:** Conceptualization, Methodology, Resources, Writing – review & editing. **Bruce L. Innis:** Conceptualization, Methodology, Writing – review & editing.

## Declaration of Competing Interest

The authors declare the following financial interests/personal relationships which may be considered as potential competing interests: Librada Fortuna, Chawanee Kerdsomboon, Vilasinee Yuwaree, Souad Mansouri and Pham Hong Thai are employed by BioNet. All other authors declare no competing interests.

## Figures and Tables

**Fig. A1:**
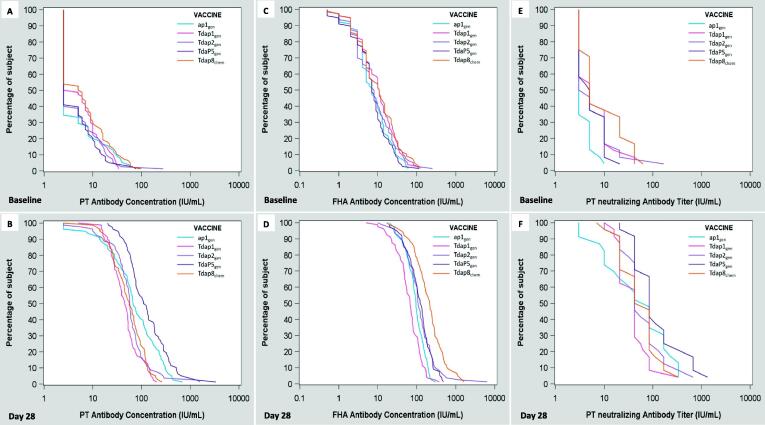
Reverse cumulative distribution curves (RCDCs) for PT-IgG, FHA-IgG and PT neutralizing antibody at baseline and 28 days post-vaccination with study group (ap1_gen_/Tdap1_gen_/Tdap2_gen_/TdaP5_gen_) and comparator group (Tdap8_chem_) in pregnant women. Panel A and B represent the RCDC for PT-IgG at baseline and day 28 post-vaccination. Panel C and D represent the RCDC for FHA-IgG at baseline and day 28 post-vaccination. Panel E and F represent the RCDC for PT neutralizing antibody titer at baseline and day 28 post-vaccination.

**Fig. A2:**
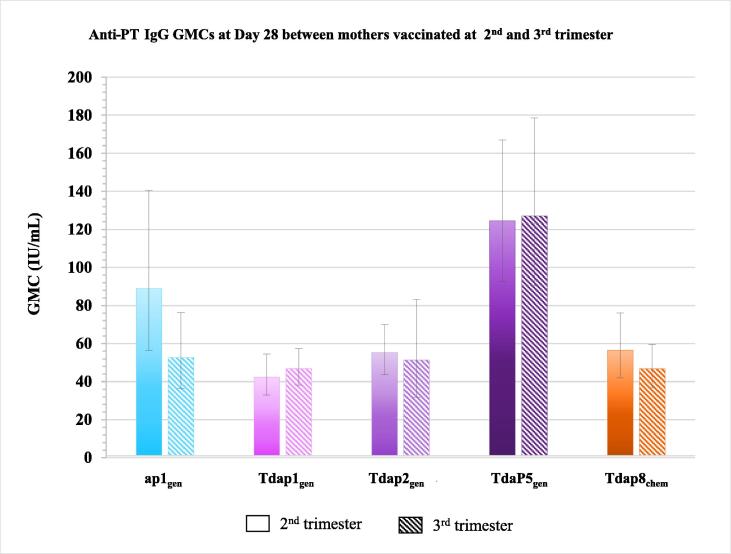
Anti-PT IgG geometric mean concentration (GMCs) at Day 28 post-vaccination between mothers vaccinated at 2^nd^ and 3^rd^ trimester of pregnancy.

**Table A1:** Selection criteria.

**Subject Characteristics and Inclusion Criteria for Pregnant Women**
A pregnant woman will be eligible for inclusion if ALL of the following criteria are met:
1. Healthy, as established by medical history, physical examination, and obstetrical risk assessment 2. Uncomplicated singleton pregnancy 3. 18–40 years of age 4. Documentation of gestational age based on last menstrual period, physical exam, and ultrasound 5. Pregnancy at ≥20 weeks gestational age but prior to 33 weeks 6. Willing and able to provide written informed consent 7. Willing and able to understand and comply with the study protocol in the principal investigator’s judgement
**Subject Characteristics and Exclusion Criteria for Pregnant Women**
Subjects with ANY of the following criteria at study entry will not be eligible for participation:
1. Any significant congenital abnormality as documented by ultrasound 2. History of significant medical illness such as immune deficiency (including HIV infection and transplant recipients), hypertension or other cardiovascular disease, or renal or hepatic diseases 3. Pregnancy complications (in the current pregnancy) such as hypertension (blood pressure [BP] >140/90 in the presence of proteinuria or BP >150/100 with or without proteinuria) or currently on an antihypertensive therapy, or pre-eclampsia 4. Endocrine disorders, including (but not limited to) hyperthyroidism, untreated hypothyroidism, and glucose intolerance (e.g., diabetes mellitus type 1 or 2) antedating pregnancy, or occurring during pregnancy and requiring interventions other than diet for control 5. Prior stillbirth or neonatal death, or multiple (≥3) spontaneous abortions 6. Prior preterm delivery <37 weeks gestation, or ongoing intervention (medical or surgical) in the current pregnancy to prevent preterm birth 7. Greater than five (5) prior deliveries 8. History of allergy to any vaccine component 9. History of neurological adverse event after injection with any vaccine 10. Having received any other vaccines within 28 days prior to recruitment (3 months for live attenuated vaccines) 11. Plan to participate in another clinical trial during the study period 12. History of receiving blood or blood component or immunoglobulin (with the exception of prophylactic anti-Rho D immune globulin) within six (6) months prior to recruitment 13. History of receiving immunosuppressive drugs or systemic corticosteroid (>0.5 mg/kg of prednisolone or equivalent for more than 14 days) within 3 months prior to recruitment 14. Having received diphtheria or tetanus or pertussis-containing vaccine or experienced a physician-diagnosed pertussis illness within 1 year prior to recruitment 15. Presence of acute febrile illness (defined as body temperature above 38°C) on the day of vaccination (This is a temporary exclusion criterion) 16. Individuals with any progressive or severe neurological disorder, seizure disorder, or with history of Guillain-Barré syndrome 17. Individuals with behavioral or cognitive impairment or psychiatric disease that, in the opinion of the investigator, may interfere with the subject's ability to participate in the study 18. Any history of smoking, alcoholism, and/or intravenous drug abuse which in the opinion of the investigator will interfere with the study outcome evaluation 19. Presence of bleeding disorders, including deep venous thrombosis or thromboembolism, or the use of anticoagulants during pregnancy, or abnormalities of splenic and thymic functions 20. Individuals with history of any illness or condition that, in the opinion of the investigator or obstetrician, would classify the pregnancy as high-risk, interfere with the results of the study, or pose additional risk to the mother or fetus/infant due to participation in the study

**Table A2:** Study vaccines.

	**Investigational vaccines**			**Licensed vaccines**	
	**Geneticallyinactivated PT (PT_gen_) based vaccines**			**PT_gen_-based reference vaccine**	**Active comparator**
	**ap_gen_** **low dose** **(ap1_gen_)^1^**	**Tdap_gen_** **low dose** **(Tdap1_gen_)^1^**	**Tdap_gen_** **reduced dose** **(Tdap2_gen_)^1^**	**TdaP_gen_** **high dose** **(TdaP5_gen_; Boostagen®)^1^^,^^3^**	**Tdap_chem_** **reduced dose** **(Tdap8_chem_; Boostrix™)^2^^,^^4^**
**Active ingredients per 0.5-mL dose**					
**TT**	–	7.5 Lf	7.5 Lf	7.5 Lf	5 Lf
**DT**	–	2.0 Lf	2.0 Lf	2.0 Lf	2.5 Lf
**PT**	1 µg PT_gen_	1 µg PT_gen_	2 µg PT_gen_	5 µg PT_gen_	8 µg PT_chem_
**FHA**	1 µg	1 µg	5 µg	5 µg	8 µg
**PRN**	–	–	–	–	2.5 µg
**Batch/Lot no.**	8005A	8006A	8004A	7007A1A	AC37B293CI
1) Manufactured by BioNet-Asia Co., Ltd. (BioNet); 2) Manufactured by GlaxoSmithKline (GSK); 3) Boostagen® is a licensed (reference) vaccine; 4) Boostrix^TM^, a licensed vaccine containing chemically inactivated pertussis toxin is the comparator to the other vaccines that all include genetically inactivated pertussis toxin. Abbreviations: TT, Tetanus Toxoid; DT, Diphtheria Toxoid; PT, Pertussis Toxoid; PT_gen_, genetically inactivated PT; PT_chem_, chemically inactivated PT; FHA, Filamentous hemagglutinin; PRN, pertactin; Lf, limit of flocculation; mL, milliliter; µg, microgram.					

**Table A3:** Anti-tetanus and anti-diphtheria IgG as assessed at baseline and 28 days after vaccination in pregnant women.

	**Anti-tetanus IgG**			**Anti-diphtheria IgG**		
	**Baseline**	**28 days after vaccination**		**Baseline**	**28 days after vaccination**	
	**GMC (IU/mL)^1^** **(95**% **CI)**	**GMC (IU/mL)^2^** **(95**% **CI)**	**Adjusted** **GMC ratio**^2^ **(95**% **CI)**	**GMC (IU/mL)^1^** **(95**% **CI)**	**GMC (IU/mL)^1^** **(95**% **CI)**	**Adjusted** **GMC ratio**^2^ **(95**% **CI)**
**Tdap1_gen_**	1.0 (0.8–1.2)	4.3 (3.8–5.0)	0.8 (0.7–1.0)	0.1 (0.1–0.1)	0.5 (0.4–0.6)	0.6 (0.4–0.8)
**Tdap2_gen_**	0.9 (0.7–1.1)	5.4 (4.6–6.4)	1.0 (0.8–1.3)	0.1 (0.1–0.1)	0.7 (0.6–0.9)	1.0 (0.7–1.3)
**TdaP5_gen_**	0.8 (0.7–1.1)	4.2 (3.6–4.8)	0.8 (0.6–1.0)^3^	0.1 (0.1–0.1)	0.8 (0.6–1.0)	0.9 (0.7–1.2)
**Tdap8_chem_**	0.8 (0.6–1.1)	5.3 (4.5–6.3)	–	0.1 (0.1–0.1)	0.9 (0.7–1.1)	–

^1^ The GMC of anti-TT and anti-DT IgG were calculated with 95% CIs at baseline and 28 days after vaccination.

^2^ The adjusted Day 28 GMC ratio between each study group (Tdap1_gen_/Tdap2_gen_/TdaP5_gen_) and comparator group Tdap8_chem_ with 95% CI were calculated based on ANCOVA model, adjusted for concentrations at baseline and subject age for anti-TT IgG analysis and adjusted for concentrations at baseline only for anti-DT IgG analysis.

^3^ The 95% CI of the adjusted GMC ratio is 0.63–0.98.

Abbreviations: CI, confidence interval; DT, diphtheria toxoid; GMC, geometric mean concentration; IgG, immunoglobulin G; IU, international unit; mL, milliliter; TT, tetanus toxoid.

**Table A4:** Proportion of pregnant women reporting local and systemic reactions within 7 days after vaccination.

	**ap1_gen_** **(N = 80)**	**Tdap1_gen_** **(N = 80)**	**Tdap2_gen_** **(N = 80)**	**TdaP5_gen_** **(N = 80)**	**Tdap8_chem_** **(N = 80)**
**At least one solicited local reaction^1^**					
Pain, % (95% CI)	72.5% (61.4–81.9)	80.0% (69.6–88.1)	80.0% (69.6–88.1)	82.5% (72.4–90.1)	88.8% (79.7–94.7)
Redness, % (95% CI)	2.5% (0.3–8.7)	1.3% (0.0–6.8)	1.3% (0.0–6.8)	3.8% (0.8–10.6)	1.3% (0.0–6.8)
Swelling, % (95% CI)	0.0% (0.0–4.5)	0.0% (0.0–4.5)	0.0% (0.0–4.5)	0.0% (0.0–4.5)	0.0% (0.0–4.5)
Induration, % (95% CI)	0.0% (0.0–4.5)	0.0% (0.0–4.5)	1.3% (0.0–6.8)	1.3% (0.0–6.8)	0.0% (0.0–4.5)
**At least one solicited systemic reaction^1^**					
Headache, % (95% CI)	8.8% (3.6–17.2)	5.0% (1.4–12.3)	21.3% (12.9–31.8)	16.3% (9.0–26.2)	15.0% (8.0–24.7)
Arthralgia, % (95% CI)	5.0% (1.4–12.3)	6.3% (2.1–14.0)	10.0% (4.4–18.8)	8.8% (3.6–17.2)	7.5% (2.8–15.6)
Chills, % (95% CI)	1.3% (0.0–6.8)	0.0% (0.0–4.5)	1.3% (0.0–6.8)	0.0% (0.0–4.5)	2.5% (0.3–8.7)
Fatigue, % (95% CI)	12.5% (6.2–21.8)	7.5% (2.8–15.6)	16.3% (9.0–26.2)	13.8% (7.1–23.3)	21.3% (12.9–31.8)
Malaise, % (95% CI)	3.8% (0.8–10.6)	5.0% (1.4–12.3)	3.8% (0.9–10.6)	5.0% (1.4–12.3)	10.0% (4.4–18.8)
Myalgia, % (95% CI)	15.0% (8.0–24.7)	25.0% (16.0–35.9)	36.3% (25.8–47.8)	35.0% (24.7–46.5)	36.3% (25.8–47.8)
Fever (≥38°C), % (95% CI)	0.0% (0.0–4.5)	1.3% (0.0–6.8)	2.5% (0.3–8.7)	1.3% (0.0–6.8)	0.0% (0.0–4.5)
Vomiting, % (95% CI)	1.3% (0.0–6.8)	0.0% (0.0–4.5)	3.8% (0.8–10.6)	3.8% (0.8–10.6)	2.5% (0.3–8.7)
Nausea, % (95% CI)	2.5% (0.3–8.7)	1.3% (0.0–6.8)	2.5% (0.3–8.7)	6.3% (2.1–14.0)	3.8% (0.8–10.6)

^1^ The pregnant woman may have one or more local or systemic reaction.

Abbreviation: CI, confidence interval.

**Table A5:** Incidence of unsolicited adverse events in pregnant women within 28 days after vaccination, by vaccine group.

**AE Summary**	**ap1_gen_**	**Tdap1_gen_**	**Tdap2_gen_**	**TdaP5_gen_**	**Tdap8_chem_**	**Total**
	**n (%), E^1^**	**n (%), E^1^**	**n (%), E^1^**	**n (%), E^1^**	**n (%), E**^1^	**n (%), E**^1^
Number of participants (N)	80	80	80	80	80	400
**Adverse events (AEs)**						
With one or more unsolicited AEs	13 (16.3), 17	17 (21.3), 27	20 (25.0), 27	13 (16.3), 19	18 (22.5), 30	81 (20.3), 120
With vaccine-related unsolicited AEs	1 (1.3), 1	3 (3.8), 3	4 (5.0), 4	2 (2.5), 2	1 (1.3), 1	11 (2.8), 11
With medically attended AE	5 (6.3), 5	4 (5.0), 4	6 (7.5), 6	4 (5.0), 4	4 (5.0), 4	23 (5.8), 23
With AE leading to withdrawal	0 (0.0) 0	0 (0.0), 0	0 (0.0), 0	0 (0.0), 0	0 (0.0), 0	0 (0.0), 0
**Serious adverse events (SAEs)**						
With one or more SAEs	0 (0.0), 0	2 (2.5), 2	1 (1.3), 1	1 (1.3), 1	1 (1.3), 1	5 (1.3), 5
With vaccine-related SAEs	0 (0.0), 0	0 (0.0), 0	0 (0.0), 0	0 (0.0), 0	0 (0.0), 0	0 (0.0), 0
With AE leading to hospitalization	0 (0.0), 0	1 (1.3), 1	1 (1.3), 1	0 (0.0), 0	1 (1.3), 1	3 (0.8), 3
With AE leading to death	0 (0.0), 0	0 (0.0), 0	0 (0.0), 0	0 (0.0), 0	0 (0.0), 0	0 (0.0), 0
With medically significant events	0 (0.0), 0	1 (1.3), 1	0 (0.0), 0	1 (1.3), 1	0 (0.0), 0	2 (0.5), 2
With SAE leading to withdrawal	0 (0.0), 0	0 (0.0), 0	0 (0.0), 0	0 (0.0), 0	0 (0.0), 0	0 (0.0), 0

^1^ The number of events (E) counts the events (AE or SAE) while number of participants (n) counts the participants who experienced the event and their proportion (%).

Abbreviations: AE, adverse event; E, number of events; n, number of participants; SAE, serious adverse event.

## Data Availability

Data will be made available on request.

## Appendix A

See Fig. A1, Fig. A2, Table A1, Table A2, Table A3, Table A4, Table A5
